# Polyketides and Meroterpenes from the Marine-Derived Fungi *Aspergillus unguis* 158SC-067 and *A. flocculosus* 01NT-1.1.5 and Their Cytotoxic and Antioxidant Activities

**DOI:** 10.3390/md19080415

**Published:** 2021-07-26

**Authors:** Cao Van Anh, Jong Soon Kang, Byeoung-Kyu Choi, Hwa-Sun Lee, Chang-Su Heo, Hee Jae Shin

**Affiliations:** 1 Marine Natural Products Chemistry Laboratory, Korea Institute of Ocean Science and Technology, 385 Haeyang-ro, Yeongdo-gu, Busan 49111, Korea; caovananh@kiost.ac.kr (C.V.A.); choibk4404@kiost.ac.kr (B.-K.C.); hwasunlee@kiost.ac.kr (H.-S.L.); science30@kiost.ac.kr (C.-S.H.); 2 Department of Marine Biotechnology, University of Science and Technology (UST), 217 Gajungro, Yuseong-gu, Daejeon 34113, Korea; 3 Laboratory Animal Resource Center, Korea Research Institute of Bioscience and Biotechnology, 30 Yeongudanjiro, Cheongju 28116, Korea; kanjon@kribb.re.kr

**Keywords:** marine-derived fungi, *Aspergillus* sp., polyketides, meroterpenes, antioxidant, cytotoxicity

## Abstract

Ten secondary metabolites, including a new grifolin analog, grifolin B (**1**); a new homovalencic acid derivative, 12-hydroxyhomovalencic acid (**7**); and a compound isolated from a natural source for the first time (**9**), along with seven known compounds, grifolin (**2**), averantin (**3**), 7-chloroaverantin (**4**), 1′-*O*-methylaverantin (**5**), 7-hydroxy-2-(2-hydroxypropyl)-5-pentylchromone (**6**), homovalencic acid (**8**), and bekeleylactone E (**10**), were isolated from two fungal strains. The structures of **1**–**10** were identified by detailed analysis and comparison of their spectroscopic data with literature values. Compounds **9** and **10** showed moderate cytotoxic activity against a panel of cancer cell lines (PC-3, HCT-15, MDA-MB-231, ACHN, NCI-H23, NUGC-3), with the GI_50_ values ranging from 1.1 µM to 3.6 µM, whereas **1** displayed a weak 1,1-diphenyl-2-picrylhydrazyl (DPPH) radical scavenging activity without cytotoxicity against all tested cell lines.

## 1. Introduction

Marine habitats have been acknowledged as prolific sources of new chemical entities with various worthwhile pharmacological activities [1]. Over the past decade, more than 1000 new marine natural products have been reported annually [2]. Whereas the discovery of new compounds from tunicates, cnidarians, and sponges is diminishing, there is a remarkable increase in the number of new substances isolated from marine-derived bacteria and fungi [2]. According to the latest statistics, new natural products (NPs) reported from marine-derived fungi accounted for almost half (47%) of the total new marine NPs reported in 2019 [2].

The genus *Aspergillus* is one of the most ubiquitous genera of filamentous fungi, and they are the major contributor to marine-derived fungal natural products [2,3]. A great number of secondary metabolites with structural diversity, such as polyketides, alkaloids, terpenes, steroids, and peptides, have been isolated from this genus, and many of them display potent biological activities [2].

**Figure 1:**
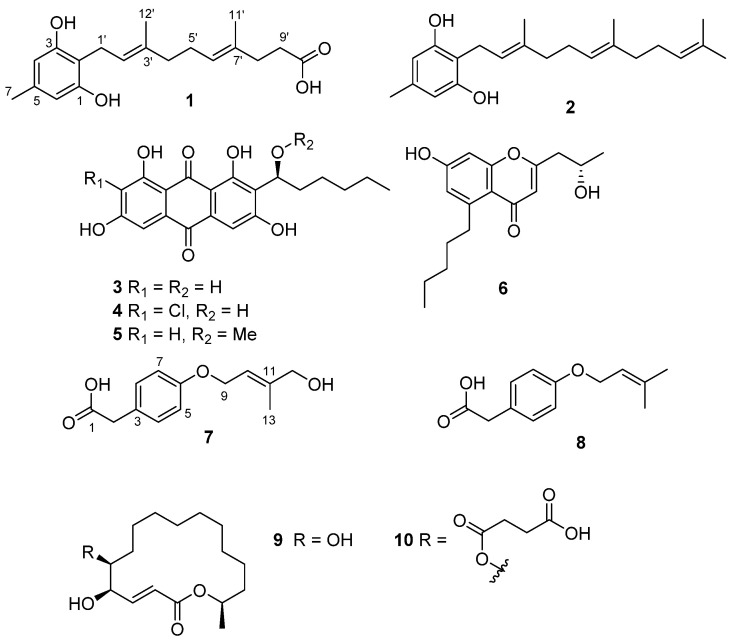
Structures of **1**–**10** isolated from *Aspergillus unguis* 158SC-067 and *A. flocculosus* 01NT-1.1.5.

As part of our ongoing program to investigate marine-derived fungi as an underexplored source of new natural products, we focused our attention on *Aspergillus unguis* 158SC-067 and *A. flocculosus* 01NT-1.1.5 strains, which showed good antimicrobial activity in the preliminary screening. Our previous studies on the EtOAc extract of *A. flocculosus* 01NT-1.1.5 grown on rice medium led to the isolation of fungal metabolites having antimicrobial properties and the suppression of RANKL-induced osteoclastogenesis activities [4,5]. To further study the secondary metabolites from marine-derived fungi, the “one strain many compounds” (OSMAC) strategy was applied by changing the culture medium from rice medium to Bennett’s broth medium. Interestingly, the ^1^H NMR spectra of the crude extracts from *A. unguis* 158SC-067 and *A. flocculosus* 01NT-1.1.5 grown in Bennett’s broth medium showed some unique peaks in aromatic and olefinic regions, which did not appear or were much smaller when cultured in the rice medium. Therefore, the extracts from two strains were chemically investigated. As a result, two new phenolic compounds (**1** and **7**), together with eight known compounds (**2**–**6** and **8**–**10**), were isolated (Figure 1). Herein, we report the isolation, structure determination, and bioactivities of these compounds.

## 2. Results and Discussion

Compound **1** was isolated as a brown solid, and its molecular formula was deduced as C_19_H_26_O_4_ by HRESIMS data (*m/z* 341.1728 [M + Na]^+^, calculated for C_19_H_26_O_4_Na 341.1724), requiring seven degrees of unsaturation. The ^1^H NMR spectrum revealed signals of two aromatic protons at *δ*_H_ 6.12 (2H, s, H-4 and H-6); two olefinic protons at *δ*_H_ 5.21 (t, *J* = 7.0, H-2′) and 5.12 (t, *J* = 7.0, H-6′); ten methylene protons at *δ*_H_ 3.24 (d, *J* = 7.1, H_2_-1′), 2.26 (m, H_2_-9′), 2.20 (m, H_2_-8′), 2.07 (dd, *J* = 7.3, 14.6, H_2_-5′), and 1.96 (t, *J* = 7.4, H_2_-4′); and three methyl groups at *δ*_H_ 2.13 (s, H_3_-7), 1.74 (s, H_3_-12′), and 1.57 (s, H_3_-11′) (Table 1). The ^13^C NMR spectrum, in combination with the gHSQC NMR spectrum, displayed nineteen resonances belonging to a carboxyl carbon at *δ*_C_ 177.9 (C-10′); six non-protonated sp^2^ carbons at *δ*_C_ 156.9 (C-1 and C-3), 137.2 (C-5), 134.6 (C-7′), 134.2 (C-3′), and 113.3 (C-2); four protonated sp^2^ carbons at *δ*_C_ 126.0 (C-6′), 125.2 (C-2′), and 108.5 (C-4 and C-6); five sp^3^ methylene carbons at *δ*_C_ 40.7 (C-4′), 35.9 (C-8′), 34.2 (C-9′), 27.5 (C-5′), and 22.9 (C-1′); and three methyls at *δ*_C_ 21.3 (C-7), 16.2 (C-12′), and 16.0 (C-11′). One carboxyl and ten sp^2^ carbons were accounted for six out of seven degrees of unsaturation, indicating that **1** possesses a monocyclic skeleton.

**Figure 2:**
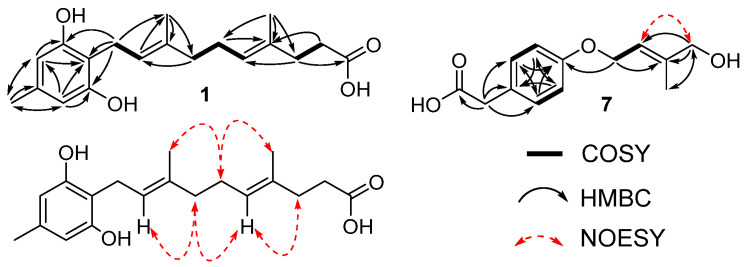
Key COSY, HMBC, and NOESY correlations for **1** and **7**.

The gross structure of **1** was identified by a detailed analysis of ^1^H-^1^H COSY and HMBC data. The structure of a symmetrical 1,2,3,5-tetrasubstituted benzene ring was identified by the HMBC cross peaks from H-4 to C-2, C-3, and C-6, and from H-6 to C-2, C-4, and C-5 (Figure 2). A methyl group attached to C-5 of the benzene ring was confirmed by the HMBC correlations from H_3_-7 to C-4, C-5, and C-6, and those of H-4/C-7 and H-6/C-7. The side chain was determined as a 4,8-dimethyldeca-4,8-dienoic acid by the COSY correlations from H_2_-1′/H-2′, H_2_-4′/H_2_-5′, H_2_-5′/H-6′, and H_2_-8′/H_2_-9′; as well as the HMBC cross peaks from H_3_-12′ to C-2′, C-3′, C-4′; from H_3_-11′ to C-6′, C-7′, C-8′; and from H_2_-8′ to C-6′ and C-10′. The side chain connected to the ring at C-2 was supported by the HMBC cross peaks from H_2_-1′ to C-1, C-2, and C-3.

The NOESY correlations from H-2′ to H_2_-4′, H_3_-12′ to H_2_-5′, and no observed correlation from H-2′ to H_3_-12′ confirmed the geometry of ∆^2′^ as 2′*E*. Similarly, ∆^6′^ was deduced as 6′*E* as shown in Figure 2. Thus, **1** is a new derivative of the co-isolated compound, grifolin (**2**) [6], and named grifolin B (Figure 1).

Compound **7** was isolated as a yellowish powder with a molecular formula of C_13_H_16_O_4_ based on its HRESIMS data (*m/z* 259.0945 [M + Na]^+^, calculated for C_13_H_16_O_4_Na 259.0946), requiring six degrees of unsaturation. The ^1^H NMR spectrum revealed the presence of seven signals, which were classified into two pairs of magnetically symmetrical protons at *δ*_H_ 7.18 (d, *J* = 8.5, H-4 and H-8) and 6.86 (d, *J* = 8.6, H-5 and H-7); an olefinic proton at *δ*_H_ 5.71 (td, *J* = 1.2, 6.3, H-10); two oxygenated sp^3^ methylenes at *δ*_H_ 4.60 (d, *J* = 6.3, H_2_-9) and 3.98 (s, H_2_-12); a sp^3^ methylene at *δ*_H_ 3.52 (s, H_2_-2); and a methyl group at *δ*_H_ 1.74 (s, H_3_-13). The ^13^C and gHSQC NMR spectra revealed the presence of thirteen carbon signals belonging to a carboxyl carbon at *δ*_C_ 176.0 (C-1); three non-protonated sp^2^ carbons at *δ*_C_ 159.2 (C-6), 140.8 (C-11), and 128.2 (C-3); two pairs of magnetically symmetrical carbons at *δ*_C_ 131.3 (C-4 and C-8) and 115.7 (C-5 and C-7); a protonated sp^2^ carbon at *δ*_C_ 121.1 (C-10); two oxygenated sp^3^ methylene carbons at *δ*_C_ 67.8 (C-12) and 65.6 (C-9); a sp^3^ methylene at *δ*_C_ 41.1 (C-2); and a methyl at *δ*_C_ 14.0 (C-13).

The structure of a symmetrical 1,4-disubstituted benzene ring was determined by the COSY correlations from H-4 to H-5 and from H-7 to H-8, and the HMBC correlations from H-4 to C-6 and C-8, from H-5 to C-3 and C-7, from H-7 to C-3 and C-5, and from H-8 to C-4 and C-6 (Figure 2). A carboxy methyl group attached to the benzene ring at C-3 was supported by the HMBC correlations from H_2_-2 to C-1, C-3, C-4, and C-8. The HMBC correlation from H_2_-9 to C-6 supported that a prenyl unit was attached to C-4 via an ether linkage. The fact that CH_2_-12 bears a hydroxy group was evidenced by the chemical shift values of H_2_-12 (*δ*_H_ 3.98) and C-12 (*δ*_C_ 67.8) as well as the molecular formula. The geometry of the double bond between C-10 and C-11 was determined as 10*E* by the strong NOESY correlation from H-10 to H_2_-12 (Figure S14). Thus, **7** is a new derivative of the co-isolated compound, homovalencic acid (**8**) [7], and named 12-hydroxyhomovalencic acid.

The previously described compounds were identified as grifolin (**2**) [6], averantin (**3**) [8], 7-chloroaverantin (**4**) [8], 1′-O-methylaverantin (**5**) [8], 7-hydroxy-2-(2-hydroxypropyl)-5-pentylchromone (**6**) [9], homovalencic acid (**8**) [7], (5*R*,6*S*,16*R*,3*E*)-5,6-dihydroxy-16-methyloxacyclohexadec-3-en-2-one (**9**) [10], and bekeleylactone E (**10**) [11] by comparison of their spectroscopic data and the signs of optical rotation with those reported in the literature. It is noteworthy that **9** was isolated for the first time from natural source in this study, and its spectroscopic data were identical to those reported for a synthetic analog by Stierle et al. (Figures S15–S18) [10].

Since some of the previously reported compounds isolated in this work have been shown to possess cytotoxic activity [8,12], **1**, **7**, **9**, and **10** were evaluated for their cytotoxicity against six cancer cell lines, HCT-15 (colon), NUGC-3 (stomach), NCI-H23 (lung) ACHN (renal), PC-3 (prostate), and MDA-MB-231 (breast), which are the most common cancer types in Korea. However, only **9** and **10** showed moderate cytotoxic activity against all of the tested cell lines, with GI_50_ values ranging from 1.1 μM to 3.6 μM (Table 2). Additionally, **1** and **7** were screened for their DPPH radical scavenging activity. Compound **1** showed a weak DPPH radical scavenging activity with an IC_50_ value of 86.4 µM, whereas **7** showed neither cytotoxic nor DPPH radical scavenging activity.

## 3. Materials and Methods

### 3.1. General Experimental Procedures

High-resolution ESIMS data were measured with a hybrid ion-trap time-of-flight mass spectrometer (Shimadzu LC/MS-IT-TOF, Kyoto, Japan). IR spectra were obtained on a JASCO FT/IR-4100 spectrophotometer (JASCO Corporation, Tokyo, Japan). The 1D and 2D NMR spectra were recorded by a Bruker 600 MHz spectrometer (Bruker BioSpin GmbH, Rheinstetten, Germany). HPLC was performed using a semi-preparative ODS column (YMC-Triart C18, 250 × 10 mm i.d, 5 µm) and an analytical ODS column (YMC-Triart C18, 250 × 4.6 mm i.d, 5 µm) (YMC Corporation, Kyoto, Japan). UV spectra were measured with a Shimadzu UV-1650PC spectrophotometer in 1 mm quartz cells (Shimadzu Corporation, Kyoto, Japan). All the reagents were purchased from Sigma-Aldrich (Merck KGaA, Darmstadt, Germany), and the organic solvents and water were distilled prior to use. Cancer cell lines were obtained from Japanese Cancer Research Resources Bank (JCRB) (NUGC-3, gastric adenocarcinoma, JCRB Cell Bank/Cat. # JCRB0822) and American Type Culture Collection (ATCC) (PC-3, prostate adenocarcinoma, ATCC/Cat. # CRL-1435; MDA-MB-231, breast adenocarcinoma, ATCC/Cat. # HTB-26; ACHN, renal adenocarcinoma, ATCC/Cat. # CRL-1611; NCI-H23, lung adenocarcinoma, ATCC/Cat. # CRL-5800; HCT-15, colorectal adenocarcinoma, ATCC/Cat. # CCL-225).

### 3.2. Fungal Material, Fermentation and Isolation of Secondary Metabolites

#### 3.2.1. Fungal Material, Fermentation, and Isolation of **1**–**6** from *Aspergillus unguis* 158SC-067

The strain *Aspergillus unguis* 158SC-067 was isolated from a seawater sample collected at the depth of 30 m near the Socheongcho Ocean Research Station, Korea, in August 2015. The fungus was identified as *Aspergillus unguis* on the basis of DNA amplification and ITS gene sequencing (GenBank accession number MZ489151). The strain was deposited in the Microbial Culture Collection, KIOST, with the name of *Aspergillus* sp. 158SC-067 under the curatorship of Hee Jae Shin.

The seed and mass cultures were conducted in Bennett’s medium (1% glucose, 0.2% tryptone, 0.1% yeast extract, 0.1% beef extract, 0.5% glycerol, natural sea salts 3.2%, and agar 1.7% for agar medium). At first, the fungus was grown on Bennett’s agar medium in a Petri dish under static condition for 7 days. Agar plugs were cut into small pieces and aseptically transferred into a 500 mL conical flask containing 300 mL of Bennett’s broth medium and placed on a rotary shaker (140 rpm) at 28 °C for 7 days for the seed culture. An aliquot (0.1% *v*/*v*) from the seed culture was inoculated into 2.0 L flasks, each containing 1.0 L of the medium, and cultured under the same conditions as described for the seed culture for 14 days. In total, 20 flasks were prepared for the mass production.

After cultivation, the culture broth and mycelium were separated by filtration. The broth was extracted with EtOAc (20 L, twice). The EtOAc layer was evaporated under reduced pressure at 37 °C to yield a broth extract (1.0 g). Afterward, the extract was separated into 10 fractions (fractions 1b–10b) by vacuum liquid chromatography on an ODS column using a stepwise elution with 100 mL each of 10%, 20%, 30%, 40%, 50%, 60%, 70%, 80%, and 90% MeOH in H_2_O and 100% MeOH. Compound **1** (3.0 mg) was isolated from fraction 7b by a semipreparative HPLC (YMC-PackODS-A, 250 × 10 mm i.d., 5 µm, flow rate 2.0 mL/min) with an isocratic elution of 60% MeOH in H_2_O for 40.0 min.

The mycelium was extracted with EtOAc (3.0 L, three times) and the EtOAc solution was evaporated under reduced pressure to yield a mycelium extract (2.0 g). The extract was fractionated into 10 fractions (fraction 1m–10m) by the same procedure described for the broth extract. Compounds **2** (1.0 mg, *t*_R_ = 54 min), **3** (10.0 mg t_R_ = 64 min), and **4** (1.0 mg, *t*_R_ = 92 min) were purified from fraction 9m by a semipreparative HPLC (YMC-PackODS-A, 250 × 10 mm i.d., 5 μm, flow rate 2.0 mL/min) with an isocratic elution of 80% MeOH in H_2_O. Fraction 10m was subjected to a semipreparative HPLC (YMC-PackODS-A, 250 × 10 mm i.d., 5 μm, flow rate 2.0 mL/min) with an isocratic elution of 90% MeOH in H_2_O to obtain compound **5** (2.0 mg, *t*_R_ = 70 min). Compound **6** (1.5 mg) was isolated from fraction 7m by an analytical HPLC (YMC-PackODS-A, 250 × 4.6 mm i.d., 5 μm, flow rate 0.8 mL/min) with an isocratic elution of 60% MeOH in H_2_O for 38 min.

#### 3.2.2. Fungal Material, Fermentation, and Isolation of **7**–**10** from *Aspergillus flocculosus* 01NT-1.1.5

*Aspergillus flocculosus* 01NT-1.1.5 was isolated from a *Stylissa* sp. sponge as previously described [4]. Based on NMR-guided isolation, the ^1^H NMR spectrum of the crude extract from the culture broth of *A. flocculosus* 01NT-1.1.5 showed some interesting peaks in olefinic and aromatic regions. Therefore, the broth extract was selected for further study. The culture broth was extracted with EtOAc, and the organic extract was fractionated into 15 fractions as described previously [13]. Compound **7** (10.0 mg) was purified from fraction 8 by a semipreparative HPLC (YMC-PackODS-A, 250 × 10 mm i.d., 5 μm, flow rate 2.5 mL/min) with an isocratic elution of 25% MeCN in H_2_O for 28.0 min. Compound **8** (10.0 mg) was isolated from fraction 10 by an analytical HPLC (YMC-PackODS-A, 250 × 4.6 mm i.d., 5 μm, flow rate 1.0 mL/min) with an isocratic elution of 50% MeCN in H_2_O for 15 min. Fraction 12 was subjected to an analytical HPLC (YMC-PackODS-A, 250 × 4.6 mm i.d., 5 μm, flow rate 1.0 mL/min) with an isocratic elution of 50% MeCN in H_2_O to yield **9** (3.0 mg, *t*_R_ = 20.0 min) and **10** (3.0 mg, *t*_R_ = 27 min).

Grifolin B (**1**): brown solid, UV (MeOH) λ_max_ (log ε) 204 (4.15), 228 (3.73), 277 (3.12) nm; IR ν_max_ 3678, 2987, 1706, 1452, 1058 cm^−1^; HRESIMS *m/z* 341.1728 [M + Na]^+^, calculated for C_19_H_26_O_4_Na 341.1724); ^1^H NMR (CD_3_OD, 600 MHz) and ^13^C NMR (CD_3_OD, 150 MHz), see Table 1.

12-Hydroxyhomovalencic acid (**7**): yellowish powder, UV (MeOH) λ_max_ (log ε) 203 (4.23), 227 (3.81), 276 (3.15) nm; IR ν_max_ 3373, 2925, 1705, 1509, 1224, 1176 cm^−1^; HRESIMS *m/z* 259.0945 [M + Na]^+^, calculated for 259.0946, C_13_H_16_O_4_Na; ^1^H NMR (CD_3_OD, 600 MHz) and ^13^C NMR (CD_3_OD, 150 MHz), see Table 1.

### 3.3. Cytotoxicity Test by SRB Assay

Cytotoxicity Test by SRB Assay has been described previously [14].

### 3.4. DPPH Radical Scavenging Assay

DPPH radical scavenging assay was performed according to the previously described method with minor modification [3,15]. The samples and a positive control, ascorbic acid, were dissolved in DMSO with final concentrations of 6.25, 12.5, 25, 50, 100, and 200 µg/mL. DPPH was dissolved in anhydrous ethanol (EtOH) with a concentration of 0.04 mg/mL. Tested samples (50 µL) were added to 50 µL of fresh DPPH, then kept in room temperature in the dark for 30 min. The optical density (OD) was measured by an AMR-100 microplate reader (Hangzhou Allsheng Instruments, Hangzhou, China) at 517 nm. The EtOH and DMSO were used as a blank and negative control, respectively. The IC_50_ values were determined by the software of GraphPad Prism 8 (GraphPad Software Inc., San Diego, CA, USA) [3].

## 4. Conclusions

In summary, on the basis of the OSMAC strategy, ten secondary metabolites, including two new phenolic derivatives (**1** and **7**), and a substance isolated from a natural source for the first time (**9**), together with seven known compounds (**2**–**6**, **8**, and **10**), were isolated from two fungal strains of the genus *Aspergillus*. Compounds **9** and **10** showed moderate cytotoxic activity, while **1** exhibited a weak DPPH radical scavenging activity without cytotoxicity. To the best of our knowledge, the known compounds (**2**–**6**) were isolated from *A. unguis* for the first time. Moreover, we also found that *A*. *flocculosus* 01NT-1.1.5 produces various chemical constituents in different culture media [4,13]. This study expanded the chemical and biological diversity of natural products isolated from marine-derived fungi. The results indicate that marine-derived fungi, particularly the *Aspergillus* genus, could be a promising source to search for bioactive natural products with unique structures for discovery of new anti-cancer drugs.

## Figures and Tables

**Table 1:** ^1^H and ^13^C NMR spectroscopic data for **1** and **7**.

Compound	1		7		
Position	*δ*_H_ (Mult, *J* in Hz)	*δ*_C_, Type	Position	*δ*_H_ (Mult, *J* in Hz)	*δ*_C_, Type
1, 3		156.9, C	1		176.0, C
2		113.3, C	2	3.52, s	41.1, CH_2_
4, 6	6.12, s	108.5, CH	3		128.2, C
5		137.2, C	4, 8	7.18, d (8.5)	131.3, CH
7	2.13, s	21.3, CH_3_	5, 7	6.86, d (8.6)	115.7, CH
1′	3.24, d (7.1)	22.9, CH_2_	6		159.2, C
2′	5.21, t (7.0)	125.2, CH	9	4.60, d (6.3)	65.6, CH_2_
3′		134.2, C	10	5.71, td (1.2, 6.3)	121.1, CH
4′	1.96, t (7.4)	40.7, CH_2_	11		140.8, C
5′	2.07, dd (7.3, 14.6)	27.5, CH_2_	12	3.98, s	67.8, CH_2_
6′	5.12, t (7.0)	126.0, CH	13	1.74, s	14.0, CH_3_
7′		134.6, C			
8′	2.20, m	35.9, CH_2_			
9′	2.26, m	34.2, CH_2_			
10′		177.9, C			
11′	1.57, s	16.0, CH_3_			
12′	1.74, s	16.2, CH_3_			

^1^H and ^13^C NMR spectra were recorded in CD_3_OD at 600 MHz and 150 MHz, respectively.

**Table 2:** Growth Inhibition (GI_50_, μM) of **9** and **10** against human cancer cell lines.

Cell Lines	9	10	Adr.
PC-3	2.7	3.6	0.17
HCT-15	3.0	2.8	0.12
MDA-MB-231	2.4	3.1	0.16
ACHN	3.4	3.1	0.16
NCI-H23	1.1	1.2	0.13
NUGC-3	2.7	2.6	0.16

Adr. Adriamycin as a positive control. GI_50_ values are the concentration corresponding to 50% growth inhibition.

## Data Availability

The data presented in the article are available in the Supplementary Materials.

## Supplementary Materials

The followings are available online at https://www.mdpi.com/article/10.3390/md19080415/s1, Figures S1–S18: the analyzed data of MS, 1D and 2D NMR spectra of compounds **1**, **7**, and **9**.
