# Supplementary material for: Polyketides and Meroterpenes from the Marine-Derived Fungi Aspergillus unguis 158SC-067 and A. flocculosus 01NT-1.1.5 and Their Cytotoxic and Antioxidant Activities
Source: Mar Drugs. 2021 Jul 26;19(8):415. doi: 10.3390/md19080415 (PMC8402063; doi:10.3390/md19080415)
Supplement: Supplementary file 1 [file marinedrugs-19-00415-s001.zip › marinedrugs-1310379-supplementary.pdf]

Polyketides and Meroterpenes from the Marine-Derived Fungi *Aspergillus unguis* 158SC-067 and *A. flocculosus* 01NT-1.1.5 and their Cytotoxic and Antioxidant Activities

Cao Van Anh [1,2], Jong Soon Kang [3], Byeoung-Kyu Choi [1], Hwa-Sun Lee [1], Chang-Su Heo [1,2] and Hee Jae Shin [1,2,*]

[1] Marine Natural Products Chemistry Laboratory, Korea Institute of Ocean Science and Technology, 385 Haeyang-ro, Yeongdo-gu, Busan 49111, Korea; caovananh@kiost.ac.kr (C.V.A.); choibk4404@kiost.ac.kr (B.-K.C.); hwasunlee@kiost.ac.kr (H.-S.L.); science30@kiost.ac.kr (C.-S.H.)

[2] Department of Marine Biotechnology, University of Science and Technology (UST), 217 Gajungro, Yuseong-gu, Daejeon 34113, Korea

[3] Laboratory Animal Resource Center, Korea Research Institute of Bioscience and Biotechnology, 30 Yeongudanjiro, Cheongju 28116, Korea; kanjon@kribb.re.kr

* Correspondence: shinhj@kiost.ac.kr; Tel.: +82-51-664-3341; Fax: +82-51-664-3340

Contents

Figure S1. HR-ESIMS data of **1**. 3

Figure S2. ^{1}H NMR data of **1** (600 MHz, CD_3OD). 4

Figure S3. ^{13}C NMR data of **1** (150 MHz, CD_3OD). 4

Figure S4. HSQC spectrum of **1** (CD_3OD). 5

Figure S5. COSY spectrum of **1** (CD_3OD). 6

Figure S6. HMBC spectrum of **1** (CD_3OD). 7

Figure S7. NOESY spectrum of **1** (CD_3OD)........ 8

Figure S8. HR-ESIMS data of **7**. 9

Figure S9. ^{1}H NMR data of **7** (600 MHz, CD_3OD). 10

Figure S10. ^{13}C NMR data of **7** (150 MHz, CD_3OD). 10

Figure S11. HSQC spectrum of **7** (CD_3OD). 11

Figure S12. COSY spectrum of **7** (CD_3OD). 12

Figure S13. HMBC spectrum of **7** (CD_3OD). 13

Figure S14. NOESY spectrum of **7** (CD_3OD)........ 14

Figure S15. LCESI-MS data of **9**........ 15

Figure S16. ^{1}H NMR data of **9** (600 MHz, CD_3OD). 16

Figure S17. ^{1}H NMR data of **9** (600 MHz, $CDCl_3$). 16

Figure S18. ^{13}C NMR data of **9** (150 MHz, CD_3OD). 17

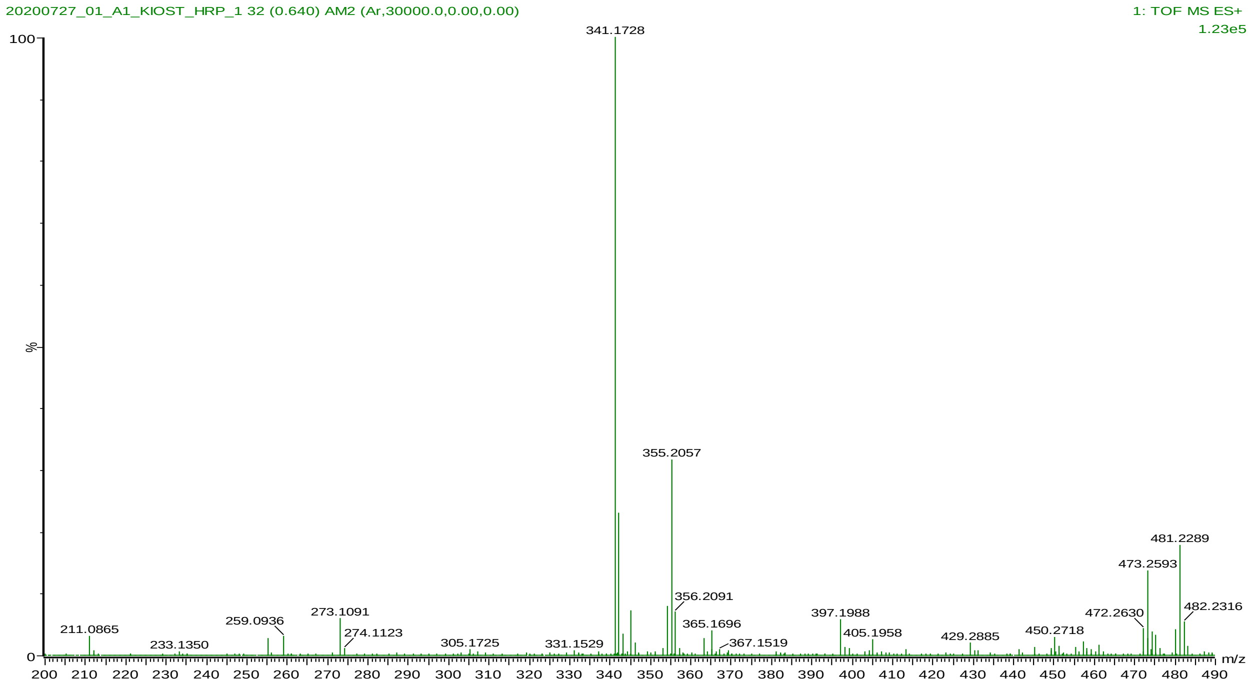

Monoisotopic Mass, Even Electron Ions
83 formula(e) evaluated with 1 results within limits (all results (up to 1000) for each mass)
Elements Used:
C: 0-50 H: 0-50 O: 0-10 Na: 0-1

Minimum:				-1.5				
Maximum:		100.0	5.0	100.0				
Mass	Calc. Mass	mDa	PPM	DBE	i-FIT	Norm	Conf(%)	Formula
341.1728	341.1729	-0.1	-0.3	6.5	650.9	n/a	n/a	C19 H26 O4 Na

Figure S1. HR-ESIMS data of **1**.

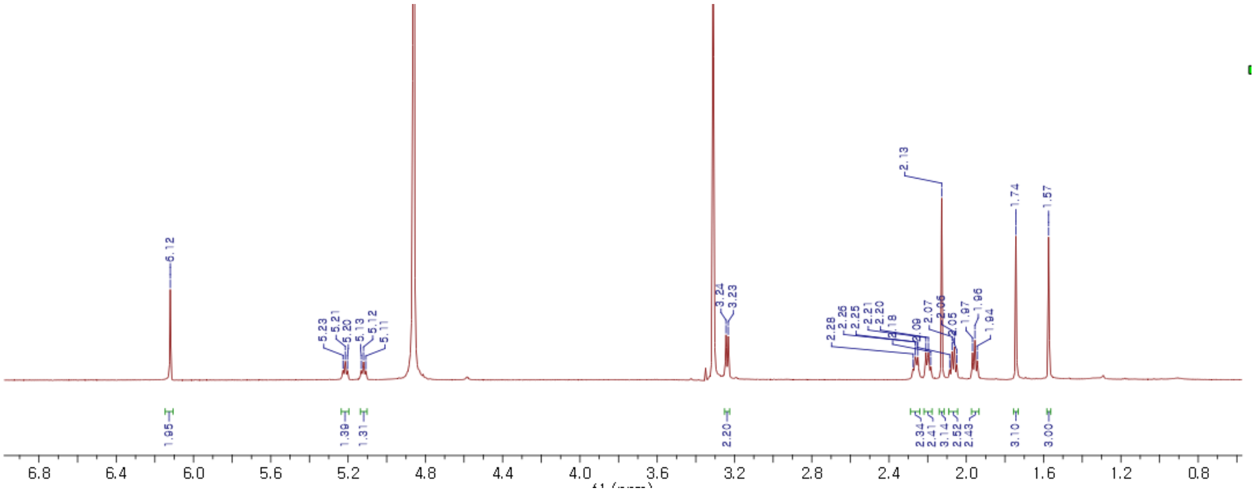

Figure S2. ^{1}H NMR data of **1** (600 MHz, CD_3OD).

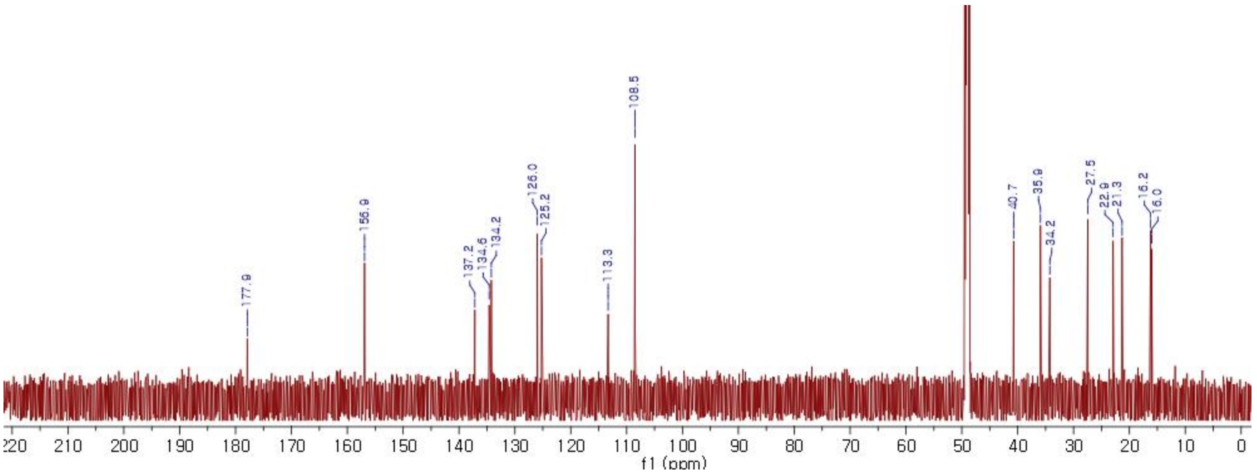

Figure S3. ^{13}C NMR data of **1** (150 MHz, CD_3OD).

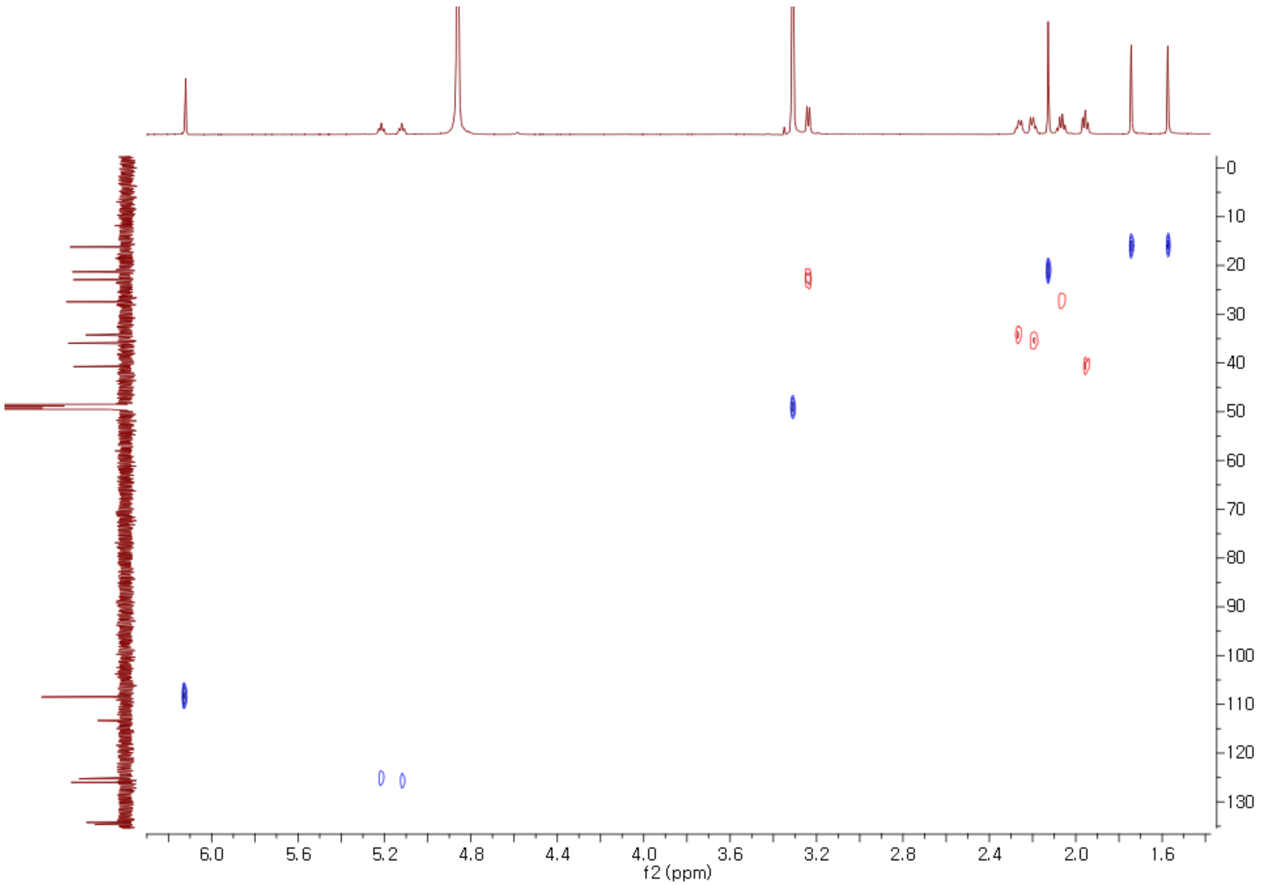

Figure S4. HSQC spectrum of **1** (CD_3OD).

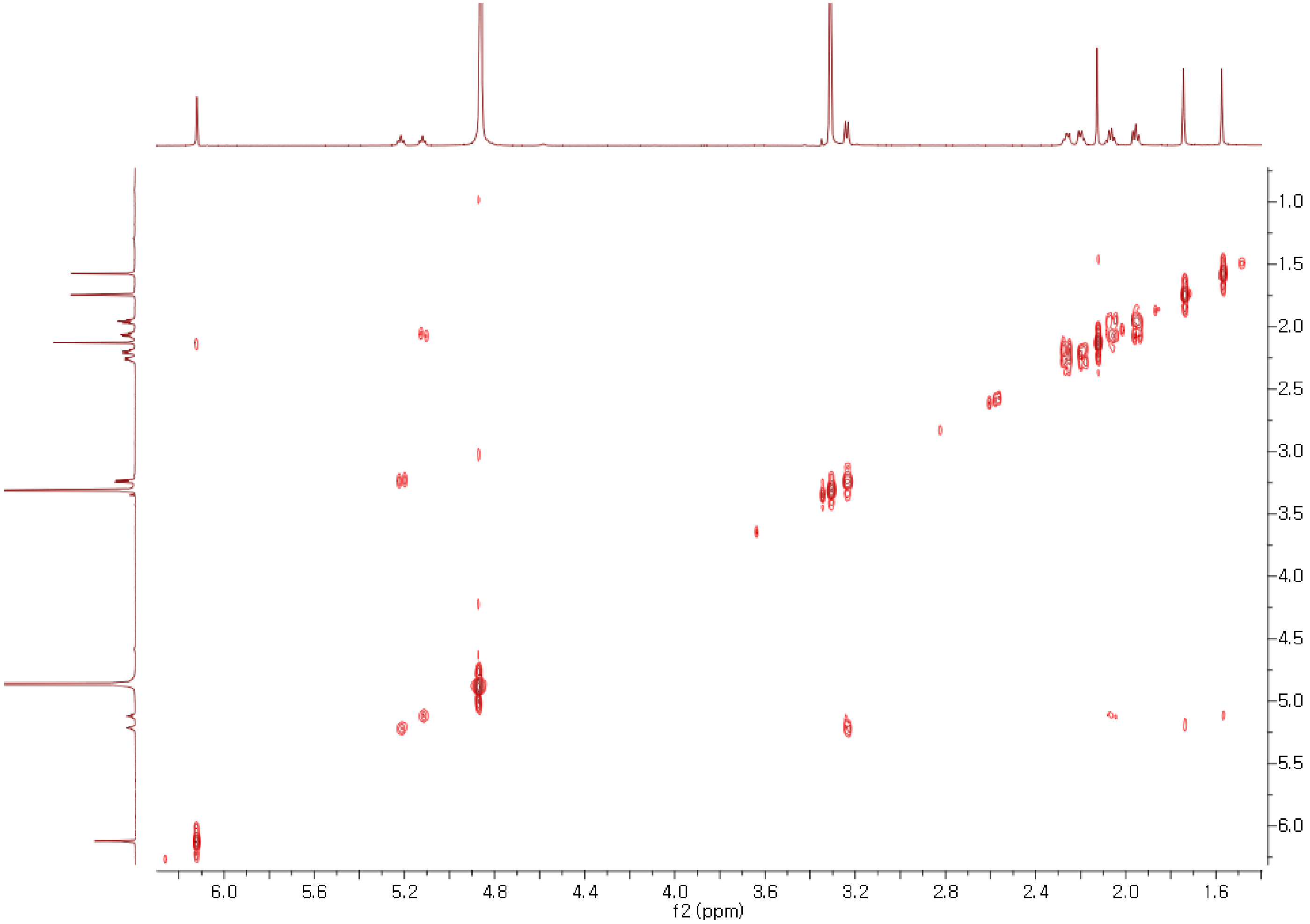

Figure S5. COSY spectrum of **1** (CD_3OD).

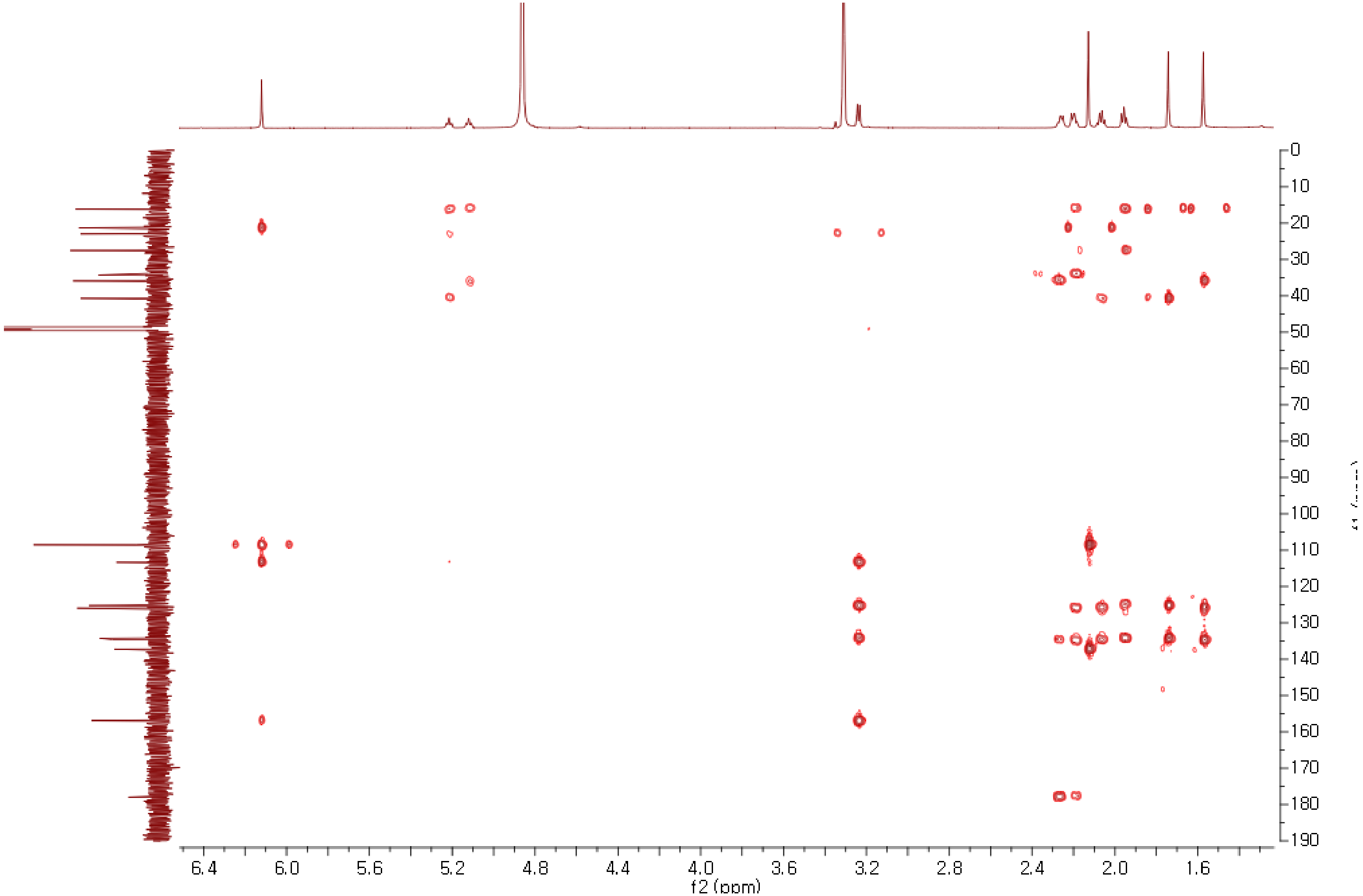

Figure S6. HMBC spectrum of **1** (CD_3OD).

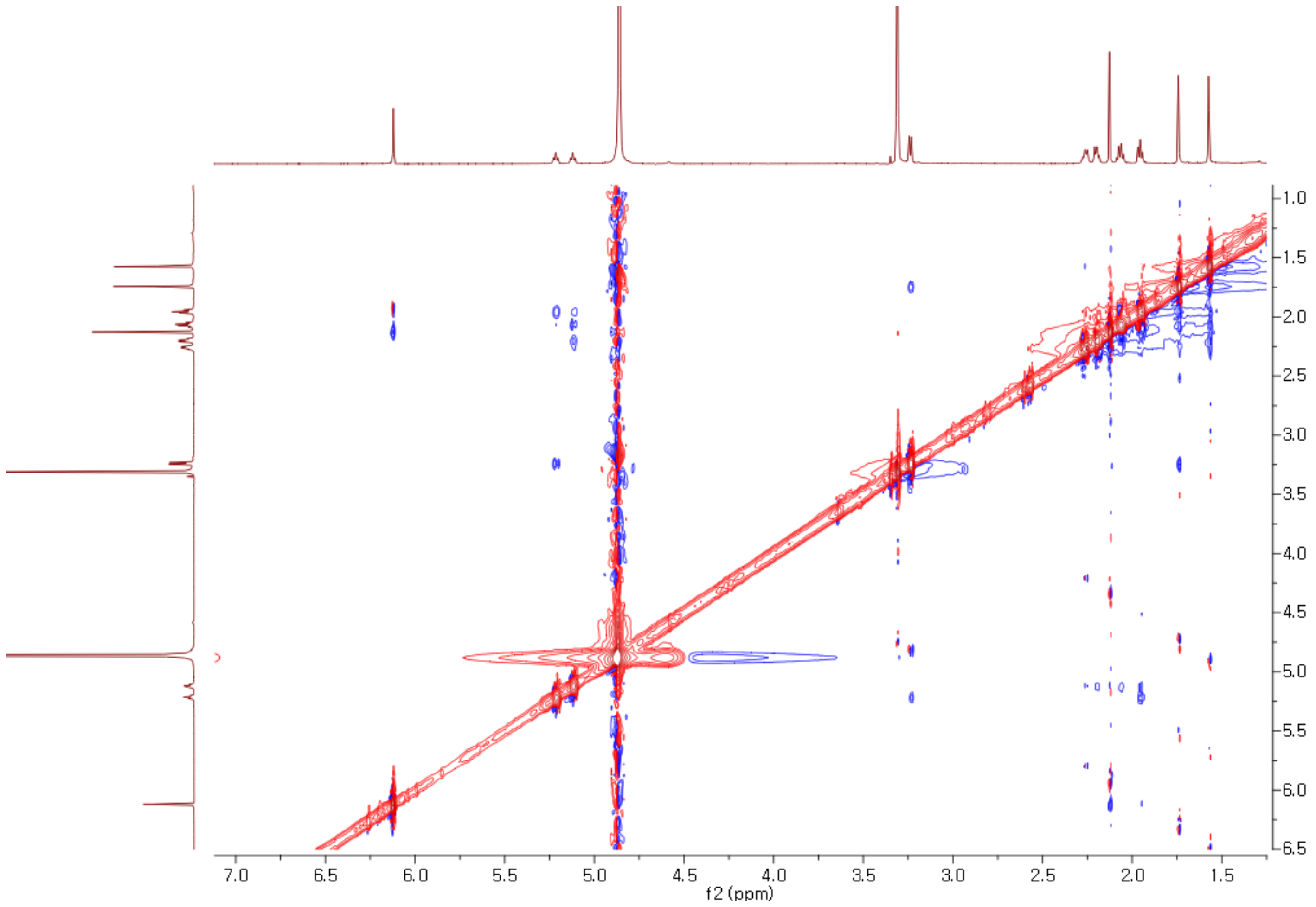

Figure S7. NOESY spectrum of **1** (CD_3OD).

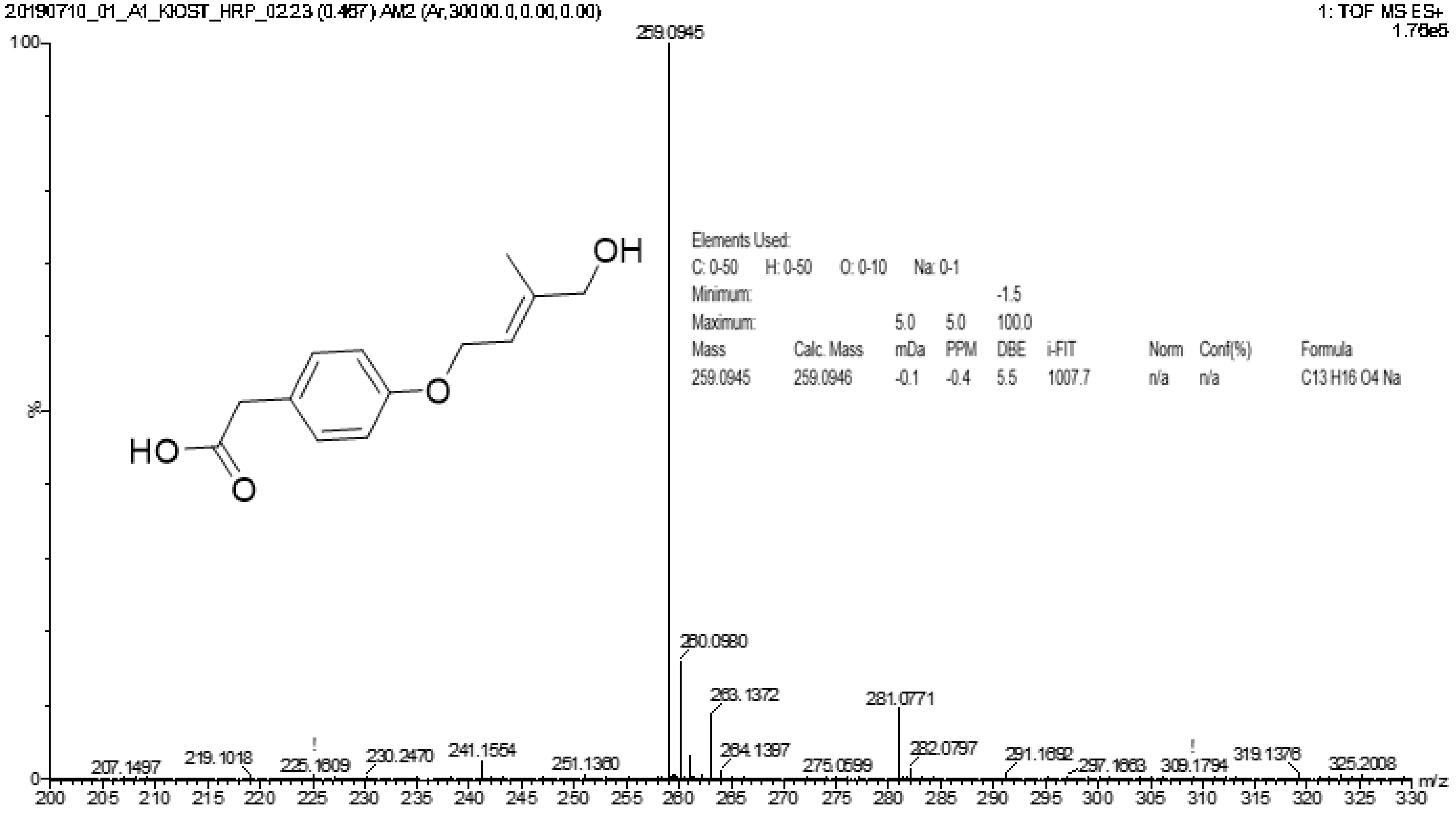

Figure S8. HR-ESIMS data of **7**.

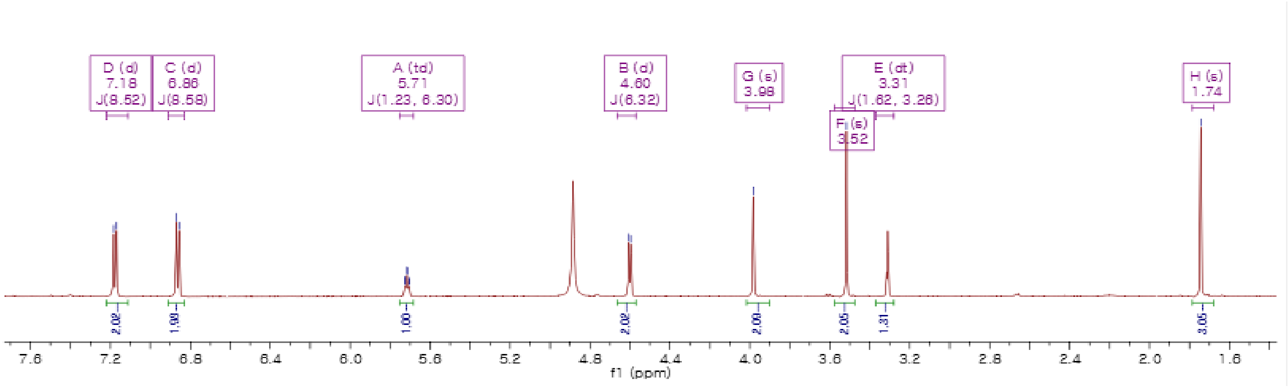

Figure S9. ^{1}H NMR data of **7** (600 MHz, CD_3OD).

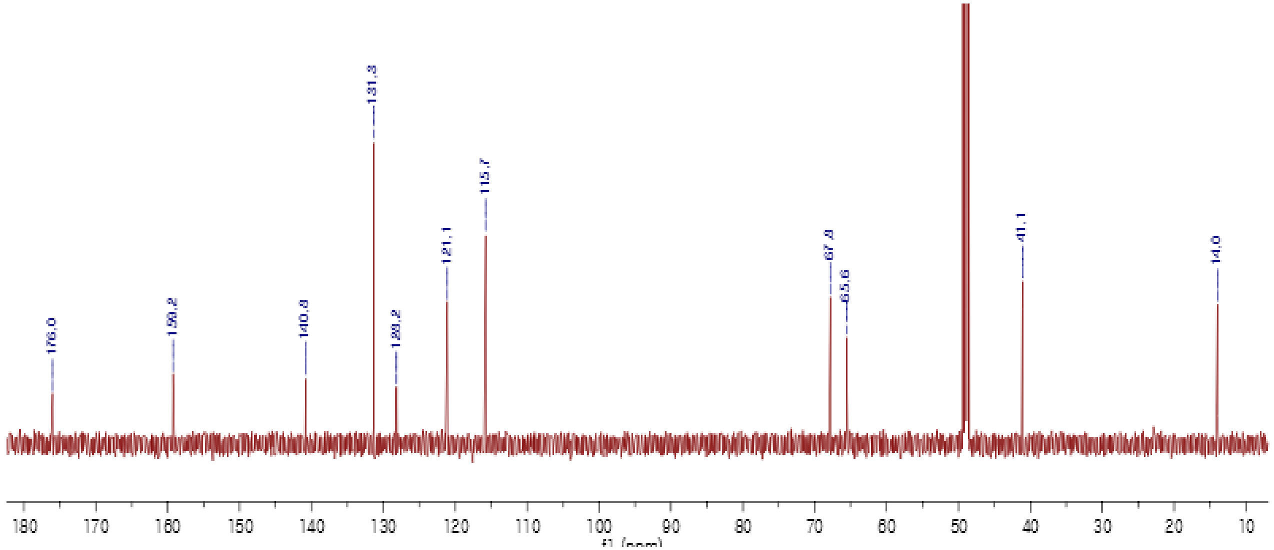

Figure S10. ^{13}C NMR data of **7** (150 MHz, CD_3OD).

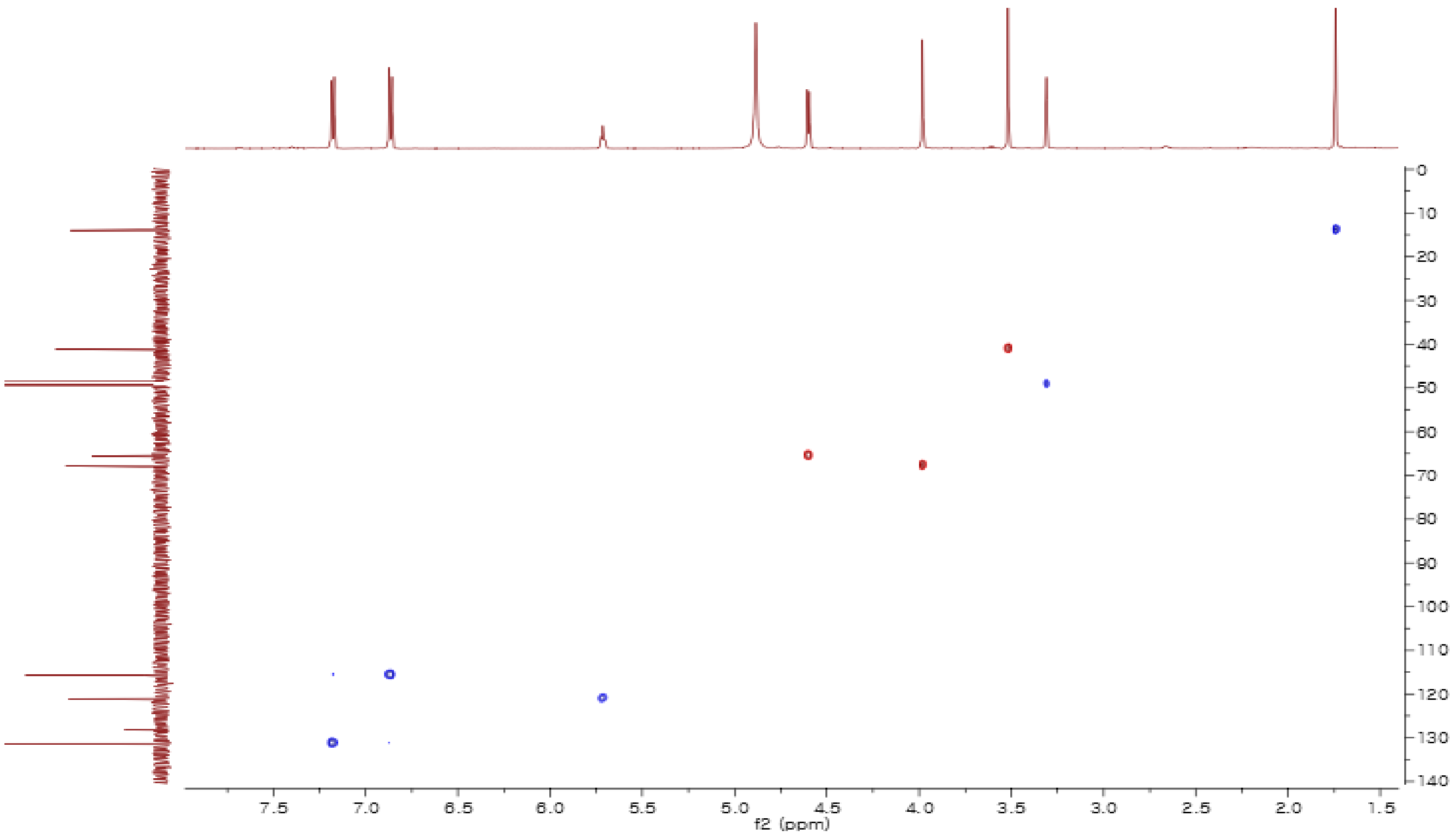

Figure S11. HSQC spectrum of **7** (CD_3OD).

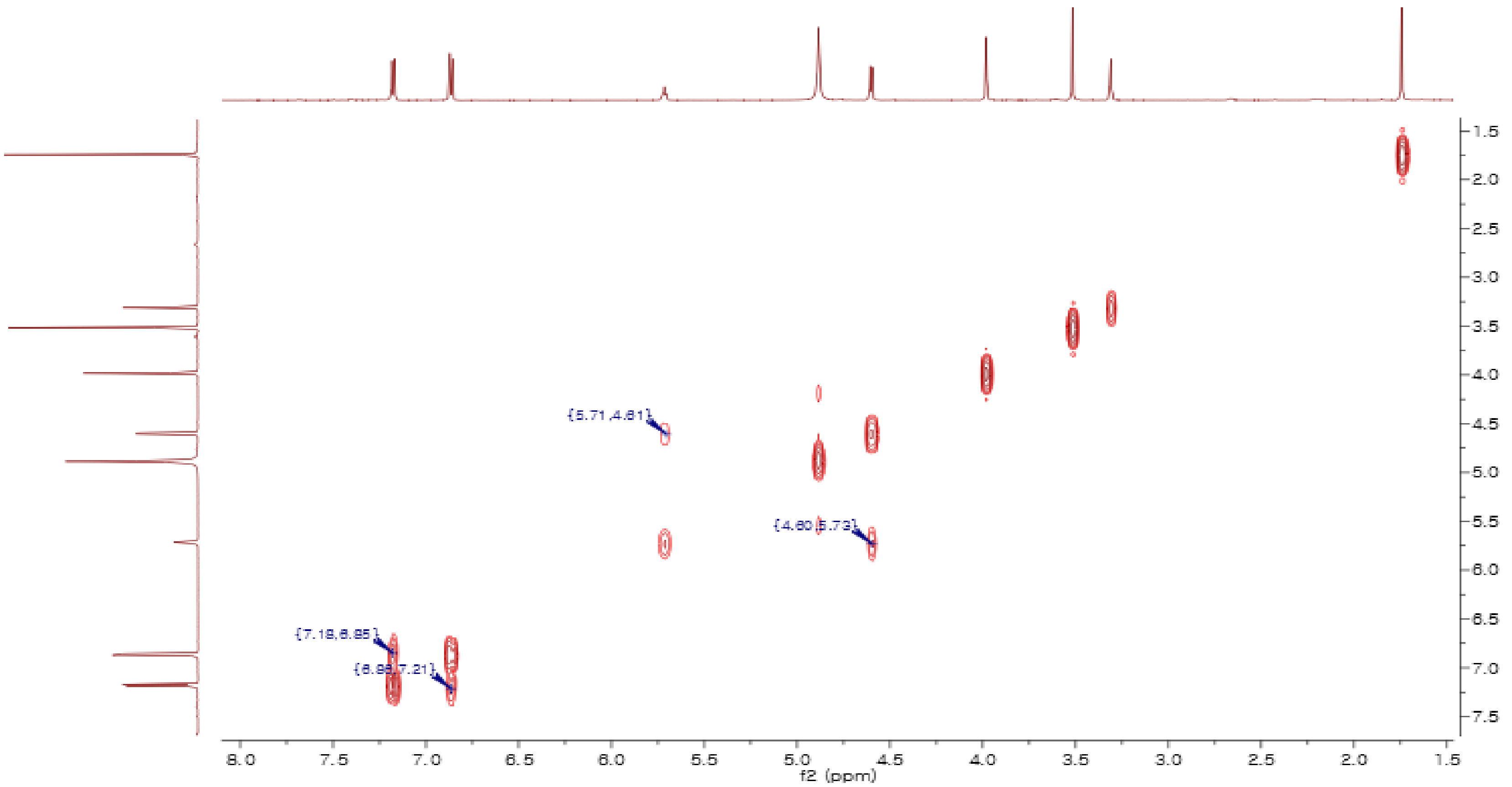

Figure S12. COSY spectrum of **7** (CD_3OD).

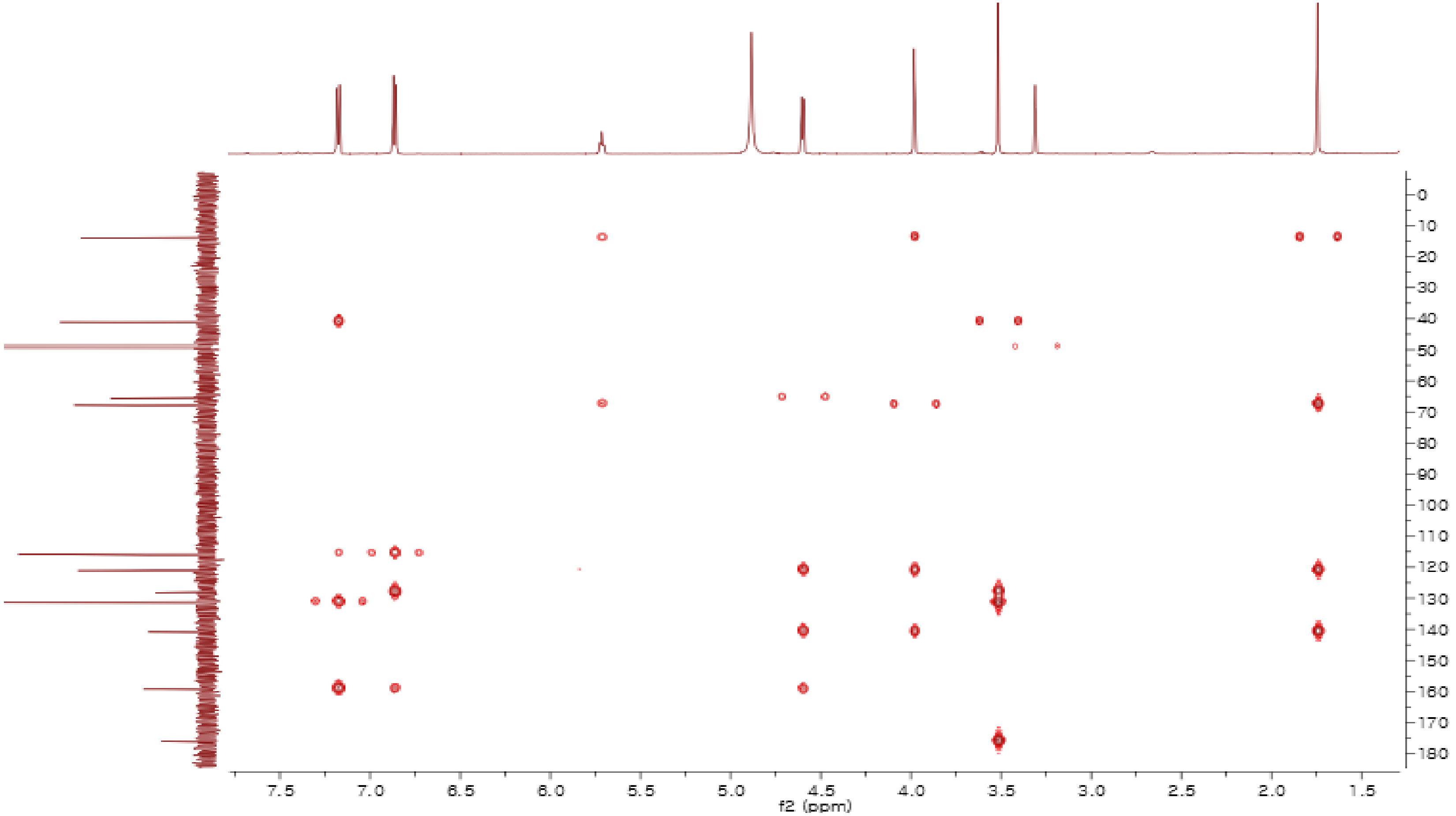

Figure S13. HMBC spectrum of **7** (CD_3OD).

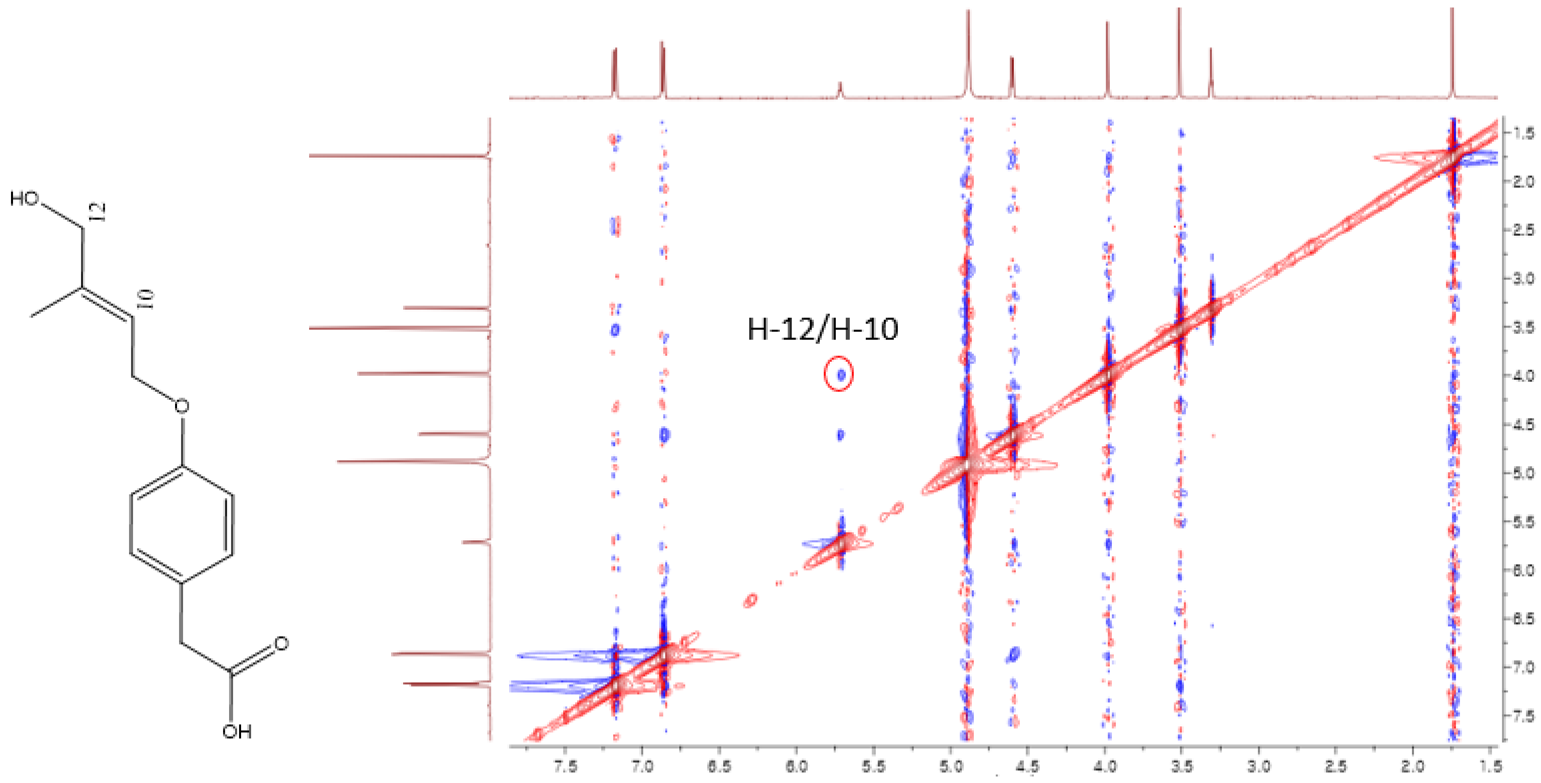

Figure S14. NOESY spectrum of **7** (CD_3OD).

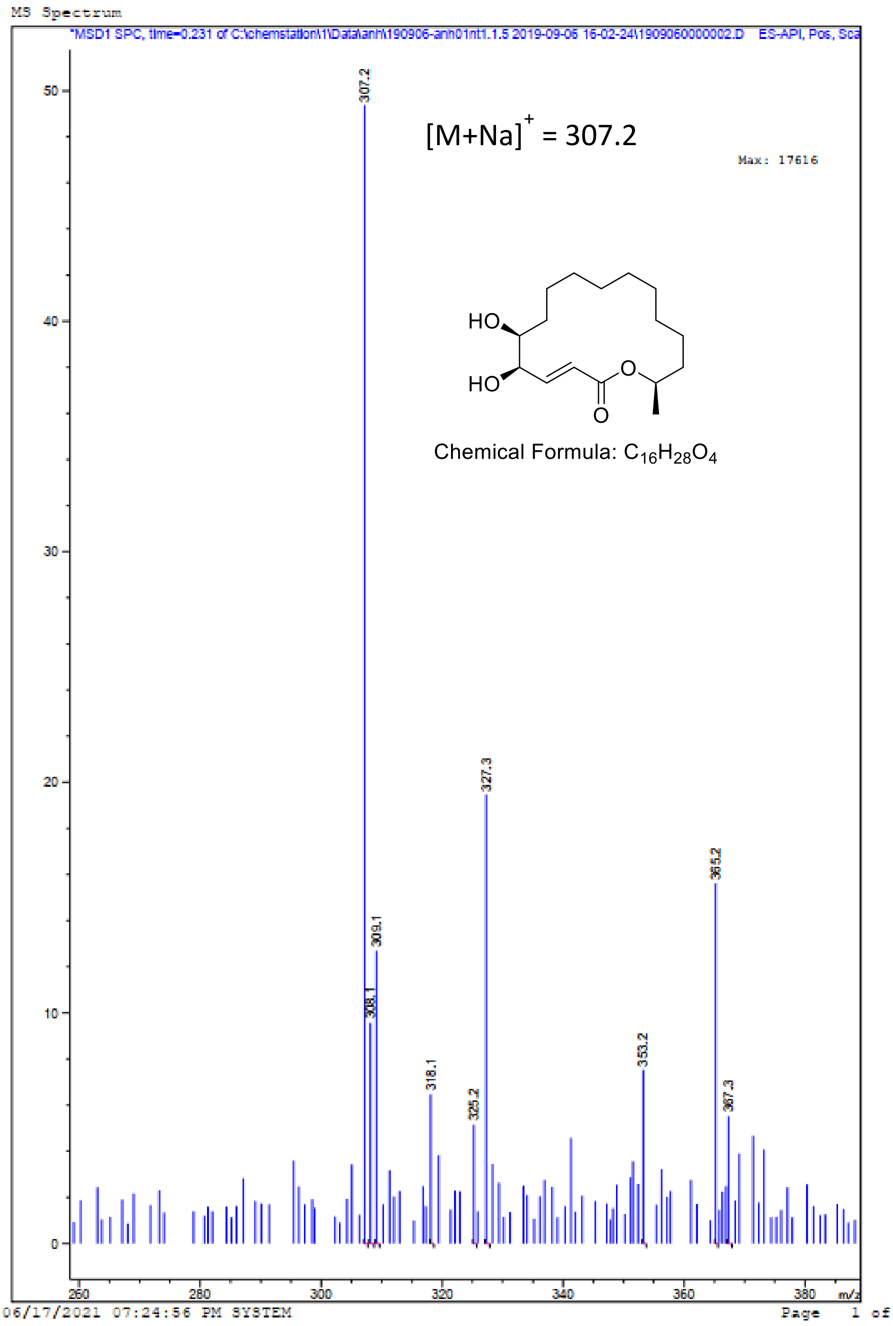

Figure S15. LCESI-MS data of **9**

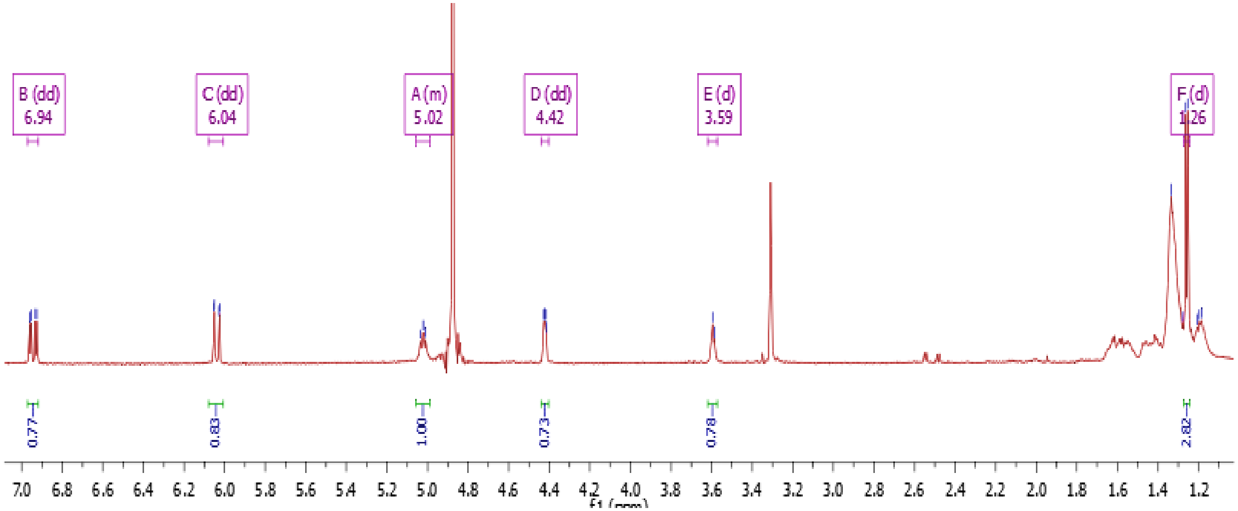

Figure S16. ^{1}H NMR data of **9** (600 MHz, CD_3OD).

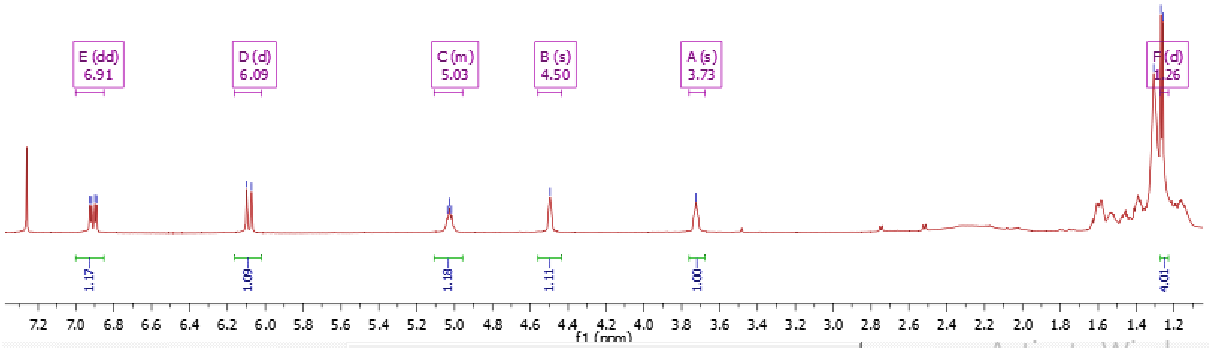

Figure S17. ^{1}H NMR data of **9** (600 MHz, $CDCl_3$).

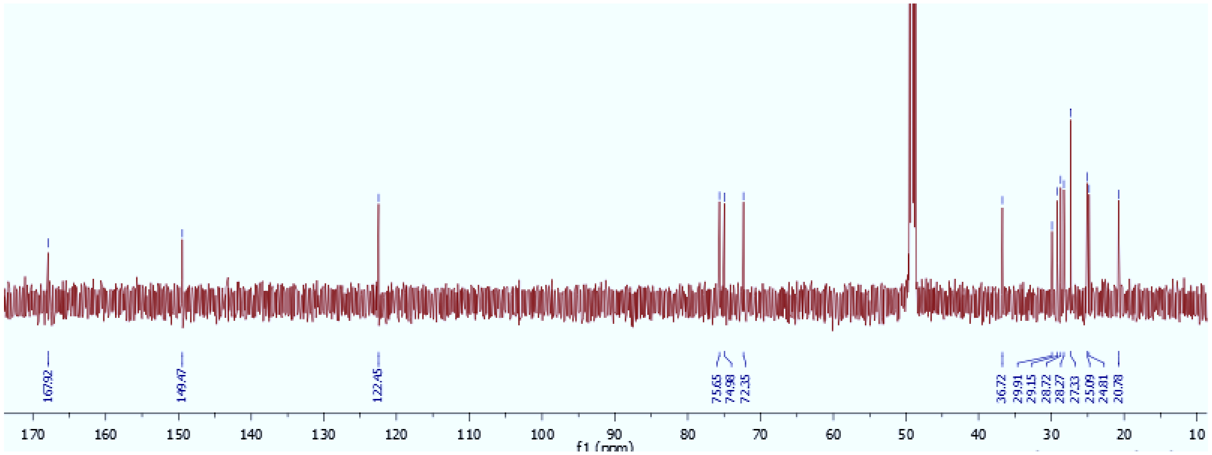

Figure S18. ^{13}C NMR data of **9** (150 MHz, CD_3OD).
